# Prevalence of Abnormal Bone-Specific Alkaline Phosphatase in Orthopaedic Trauma Patients: A Cross-Sectional Study From a Tertiary Trauma Centre

**DOI:** 10.7759/cureus.24264

**Published:** 2022-04-18

**Authors:** Devarshi Rastogi, Nayank Gautam, Zeenat Ara, Shah Waliullah, Rajeshwar N Srivastava

**Affiliations:** 1 Orthopaedic Surgery, King Georges Medical University, Lucknow, IND; 2 Orthopaedic Surgery, King Georges Medical Unversity, Lucknow, IND

**Keywords:** strobe, total alkaline phosphatase (tap), osteoblasts, glycoprotein, alkaline phosphatase (alp), balp (bone specific alkaline phosphatase), osteoporotic

## Abstract

Introduction: Bone-specific alkaline phosphatase (BALP) has been a sensitive and reliable marker of bone metabolic activity. The study aimed to study the epidemiology of orthopaedic trauma patients and estimate the level of BALP in these patients.

Material and methods: This hospital-based observational cross-sectional study was conducted on over 300 patients admitted to the orthopaedic trauma unit in the level I trauma centre of North India during the study period from January 2020 to January 2021. The level of BALP was assessed in serum samples of 258 patients.

Result: A total of 300 patients were included in this study, with an average age of 33.52±17.16 years. Road traffic accidents were the most common mode of injury among admitted patients. We found a significant linear correlation between age and mean BALP levels (P-value < 0.005). The mean age of patients having abnormal BALP is 63.0±16.61 years. Females (22.61±16.23) had greater mean BALP levels than males (13.15±5.86). Among different fractures, fractures around the hip had a higher mean BALP level (23.17±15.07). Patients with diabetes mellitus have higher mean BALP levels.

Conclusion: BALP is a cost-effective, readily available bone marker to assess the metabolic activity of orthopaedic trauma patients. Old age patients should undergo routine BALP estimation at admission to rule out metabolic disorders, and timely intervention will prevent complications associated with metabolic disorders.

## Introduction

Traumatic injuries are relatively prevalent around the globe. Traumatic orthopaedic injuries account for the majority of them [1]. Trauma places a significant strain on the country's health care system and economy. Trauma is responsible for 9% of global mortality and threatens the global healthcare system [2]. In southeast Asian countries, road traffic accidents are among the top five causes of morbidity and mortality [3]. With the increase in automobile accidents, deaths have risen substantially. India has a fatality rate of 10.5 per 10,000 automobile accidents, compared to <2 per 10,000 in developed nations [4]. One of the main reasons for the rise in orthopaedic injuries in the elderly population is osteoporosis. Osteoporosis affects roughly 200 million people, and 8.9 million fractures are osteoporotic [5]. A better understanding of the epidemiology of trauma can be of great help in planning preventive and curative strategies.

Bone-specific alkaline phosphatase (BALP) is a bone-specific isoform of alkaline phosphatase (ALP). BALP is a glycoprotein present on the surface of osteoblasts that indicates the biosynthesis activity of these bone-forming cells. It is a member of the family of zinc metalloproteins and works in an alkaline environment. BALP has been proven to be a sensitive and reliable bone metabolism measure. Total alkaline phosphatase (TAP) is a routinely used marker that is a valuable index of bone formation in subjects with normal liver function [6]. BALP is a good biochemical marker for bone formation in bone diseases and osteoporosis. Several studies have suggested that quantification of BALP in serum may be a superior index of bone formation compared to TAP [7,8].

This work sought to study the epidemiology of traumatic orthopaedic patients admitted to level I trauma centre in north India and estimate the level of BALP in these patients.

## Materials and methods

The present work was a hospital-based observational cross-sectional study reported under the Strengthening the Reporting of Observational Studies in Epidemiology (STROBE) guidelines [9]. The study was conducted on 300 orthopaedic trauma patients admitted to the level I trauma centre in North India, following the ethical guidelines of the Declaration of Helsinki during the study period between January 2020 and January 2021.

All orthopaedic trauma patients admitted to the level I trauma centre within seven days of injury were enrolled in this study. Laurer et al. [10] observed a decrease in BALP enzyme activity in trauma patients and it reaches a minimum within the first week of trauma, then afterwards activity increases. Considering these findings, cut-off points of seven days were taken. BALP was assessed in patients over 18 years of age. Patients below 18 years of age were excluded as BALP levels are higher in skeletally immature patients due to higher osteoblastic activity in this age group [10,11]. Patients requiring emergency neuro-surgical or trauma surgical intervention were excluded. A detailed demographic and history of the event, trauma mechanism, and clinical examination were conducted on all the patients. Written informed consent had already been collected from the patients or their attendants at the time of presentation for future research activities. After resuscitation in the emergency department, the patients were shifted to the orthopaedic trauma unit for further management. We divided the injuries into upper limb fractures, lower limb fractures, thorax, spine, and pelvis injuries (Table 1).

**Table 1:** Distribution of patients on the basis of their body region involved in injury body region involved

Body region involved	Frequency	Percentage (%)
Right upper limb	43	14.3
Left upper limb	34	11.3
Right lower limb	149	49.7
Left lower limb	101	33.7
Thorax	5	1.7
Spine	32	10.7
Pelvis	16	5.3

The principal investigator and one of the co-investigators independently reviewed all patient records to assess the reliability of the data collection method.

Five millilitres of peripheral venous blood were collected from each patient in a red plain vial at admission. After centrifugation, each serum sample was pipetted into plastic Eppendorf tubes (Eppendorf, Hamburg, Germany) for evaluation of BALP and stored at −80 °C until assay. Samples were prevented from repeated freeze/thaw cycles. All samples and reagents were brought to room temperature before performing the assay. Standard calibration curves were plotted, and corresponding concentrations were determined based on mean absorbance values. The level of BALP was assessed in serum samples from all the subjects. This was measured by an enzyme-linked immunosorbent assay (ELISA) as per manufacturer protocol.

Statistical analysis

Data were analyzed with the Statistical Package for Social Sciences, version 23 (SPSS Inc., Chicago, IL). Discrete (categorical) data were summarized using proportions and percentages (%) while quantitative data were summarized as mean (SD), median, or mode. Chi-square and other appropriate tests were used to check associations. For continuous variables, means were compared using the one-way analysis of variance. The level of statistical significance was set at P < 0.05.

## Results

This study included a total of 300 patients, with an average age of 33.52±17.16 years. The majority of the patients in this study were male, 84.7%, with 15.3% being female. There were 226 non-alcoholics (75.3%) and 74 alcoholics (24.7%) among the participants. Road traffic accidents were the most common cause of injury, accounting for 71.3% (214) of cases, followed by falls from height, accounting for 16.7% (50) of patients, slip-on ground/simple fall accounting for 7.7% (23) of cases, assault accounting for 2% (6), firearm injuries accounting for 1.3% (4) of cases, and crush injuries accounting for 1% (3) of patients. Only 29.9% of RTA patients wear safety equipment such as helmets and seat belts. Among the 214 RTA patients, 46.2% were drivers, 44.1% were pillion riders/co-passengers, and 12.6% were pedestrians. Forty-eight percent (144) of cases presented within one day of injury rest showed late. In this study, the majority of patients (233, 77.6%) underwent surgical treatment, 44 (14.7%) received conservative treatment, and 23 (7.7%) received both types of treatment (Table 2).

**Table 2:** Characteristics of the included patients in the present study

Mean age (in years)	33.52±17.16
Gender	
Male	84.70%
Female	5.30%
Use of safety measures	29.90%
Alcohol intake	24.70%
Driver	46.20%
Pedestrian	12.60%
Pillion rider	44.10%
Comorbidities	
Diabetes mellitus	11.00%
Hypertension	13.30%
Open/closed fractures	
Open	33.70%
Closed	54.60%
Both	11.70%
Type of management	
Conservative	14.70%
Surgical	77.60%
Both	7.70%

In this study, we looked at 258 individuals over the age of 18 and examined their BALP levels. The level of BALP was found to rise with age (Table 3).

**Table 3:** Correlation of patients on the basis of their age and bone-specific alkaline phosphatase (only for more than 18 years of age) *P-value <0.05 was significant.

Age (in years)	N=258	Bone-specific alkaline phosphatase		P-value*
		Mean±SD	Min.–Max.	
18–38	159	10.73±3.79	5.40–29.20	<0.05
39–59	65	16.35±4.22	8.60–34.10	
≥60	34	29.76±14.93	9.15–74.20	

**Figure 1:**
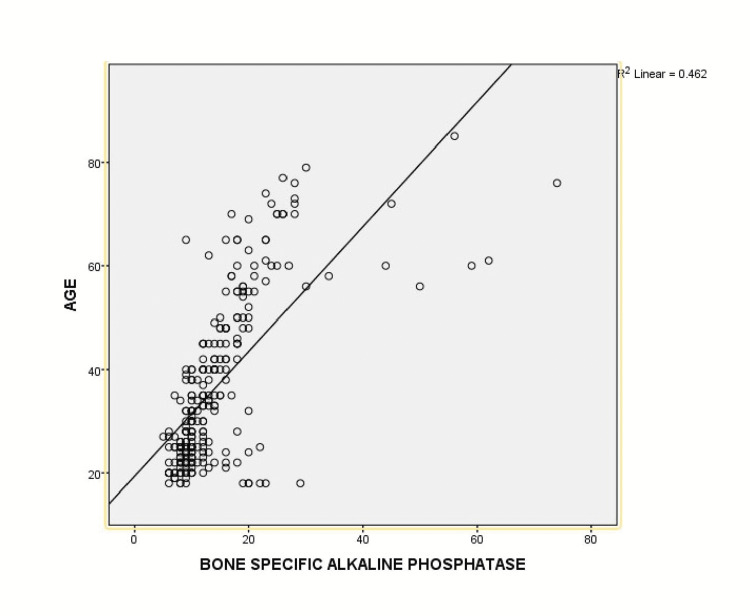
There is a positive correlation between age and bone alkaline phosphatase level. As the age increase, there is an increase in BALP level.

We found a significant correlation between age and mean BALP levels (Figure 1).

Out of 258 patients, 31 patients had BALP levels higher than the standard value (22 mcg/L), with a mean of 32.86±13.75. Among 31 patients, 14 were females, with a mean BALP of 39.94±16.93, and 17 were males, with a mean BALP of 27.04±6.48. The mean age of patients having abnormal BALP is 63.0±16.61years, with the mean age of females being 65.93±9.04 years and the mean age of males being 60.59±20.91 years. Females (22.61±16.23) had more significant mean bone alkaline phosphatase levels than men (13.15±5.86) in this study (Table 4).

**Table 4:** Distribution of patients on the basis of higher bone-specific alkaline phosphatase (BALP>22)

Gender	N	BSAP mean±SD	Minimum	Maximum	P-value
Female	14	39.94±16.93	23.10	74.20	0.007
Male	17	27.04±6.48	22.10	50.30	
Total	31	32.86±13.75	22.10	74.20	

Gender and mean BALP have shown a statistically significant relationship. In this study, the slip-on ground had a higher mean BALP level (30.88±17.09) than other forms of the mode of trauma and was statistically significant. Patients with fractures around the hip (fractured neck of femur, intertrochanteric fracture femur, subtrochanteric fracture femur) had a higher mean BALP level (23.17±15.07) as compared to other bone fractures and were statistically significant. Patients with diabetes mellitus also have higher mean BALP levels.

## Discussion

Traumatic injuries are posing substantial and growing difficulties to healthcare systems today [12]. Traumatic injuries are one of the leading causes of death worldwide, according to the World Health Organization (WHO), with 90% of estimated injuries happening in poor and middle-income countries [13]. Orthopaedic injuries account for a high percentage of traumatic injuries.

The total number of patients in our study was 300, and we discovered that the majority of them were young male adults, with a mean age of 33.52±17.16 years. Similarly, Manwan et al. [1] observed that the average age of patients with severe orthopaedic injuries was 33.5 years. The majority of patients hospitalized were men, with a sex ratio of 7:3. Similarly, Soleymanha et al. [14] found a mean age of 34.5 years, with men (75.1%) outnumbering females (24.9%), which is consistent with our data. Young men are more likely to engage in outdoor activities, explaining the age and gender disparity.

Our study found that RTA was the most prevalent cause of injury, accounting for 71.3% of cases, followed by falls, accounting for 16.7% of cases. Similarly, Rastogi et al. [15] observed that roadside injuries were the most frequent (66.71%) cause of injuries, followed by household injuries (23.66%).

Only 29.9% of patients used safety measures such as helmets and seatbelts in our study; similarly, Chaudhary et al. [16] showed that only 14% of injured patients used helmets while driving two-wheelers while 30% of those injured in four-wheelers used seatbelts. This is because of a lack of understanding and inadequate enforcement of traffic laws in underdeveloped countries. We observed that 48% of patients presented to the hospital within 24 hours of injury, and the rest of the patients showed up late. In a study of orthopaedic trauma among patients attending the accident and emergency department in a tertiary care hospital, Rohilla et al. [17] discovered that roughly 23.18% of RTA victims were pedestrians, and 67.6% arrived at the hospital within 12 hours of injury. Our study found that most patients presented late because our centre is a tertiary care centre, and patients are referred from different hospitals and remote areas.

Out of 300 patients, BALP levels were assessed in serum samples of only 258 patients. In a cross-sectional investigation, Yessayan et al. [18] showed that when BALP>22 ng/ml, all participants exhibited mild/moderate bone turnover characteristics, and BALP is the best predictor of biopsy results, with an appropriate cut-off of 22 ng/ml. There were 31 (12%) individuals in our research with a BALP of more than 22 ng/ml and a mean BALP of 32.86±13.75. The average age of the 31 patients with greater BALP was 63.01±6.61 years. In our study, the mean bone alkaline phosphatase level increased with age, and we found a highly significant association between age and mean BALP. Higher BALP levels were observed in old age due to osteoporosis and various other metabolic disorders [19]. Mean BALP levels were higher in females than in males, indicating a link between gender and mean BALP. A possible explanation for higher levels of BALP among females is due to postmenopausal osteoporosis [20]. Garnero and Delmas [21] used a new immunoradiometric assay to measure BALP in patients with metabolic bone disease. They found that BALP increased linearly with age in both sexes, but the mean BALP serum levels were not significantly different between men and women. A higher mean BALP level was observed in injuries like slip-on ground compared to other modes of injury; it may be because of underlying metabolic bone disease. Krappinger et al. [22] found low-energy osteoporotic fractures occurring in patients >65 years after simple falls. Our study also found that the pattern of bone involvement has a significant correlation with BALP; a high mean BALP was observed in fractures around the hip (fractured neck of femur, intertrochanteric fracture femur).

Chen et al. [23] compared the relationship between BALP and bone mineral density (BMD) in diabetic and non-diabetic patients to determine the risk of fracture in diabetic patients and found that BALP and BMD are inversely related in both DM and non-DM patients. This study found a strong correlation between diabetes mellitus and BALP. After correcting for other variables (tobacco smoking, alcohol use, physical activity, glycosylated haemoglobin), BALP is substantially linked with BMD, suggesting that BALP may interact with other factors affecting bone metabolism in diabetics.

The major limitation of our study is that it was a single-centric study, so the results cannot be generalized. Due to its cross-sectional nature, we could not comment on sequential changes in BALP level with time after injury. We had not included evaluation of bone mineral density and other metabolic tests in affected individuals in the present study, so we could not comment on the exact reason for the elevation of BALP level. Further multicentric, prospective studies with the inclusion of metabolic workup can generate robust data.

## Conclusions

Young adults are more prone to RTA, and effective government policy implementation is a need of the hour to prevent RTA. Advancing age, diabetes, and poor bone quality are significant risk factors associated with the rise in BALP. BALP is a cost-effective, readily available bone marker to assess the metabolic activity of orthopaedic trauma patients. Older patients should undergo routine BALP estimation at admission to rule out metabolic disorders, and timely intervention will prevent complications associated with metabolic disorders.

## References

[1] Manwana ME, Mokone GG, Kebaetse M, Young T (2018). Epidemiology of traumatic orthopaedic injuries at Princess Marina Hospital, Botswana. SA Orthop J.

[2] Wui LW, Shaun GE, Ramalingam G, Wai KM (2014). Epidemiology of trauma in an acute care hospital in Singapore. J Emerg Trauma Shock.

[3] Paden M, McGee K, Krug E, Injury Injury (2022). A leading cause of the global burden of disease. https://www.who.int/publications/i/item/injury-a-leading-cause-of-the-global-burden-of-disease-2000.

[4] (2022). Capacity building for developing trauma care facilities on National Highways Operational Guidelines. https://dghs.gov.in/WriteReadData/userfiles/file/Operational_Guidelines_Trauma.pdf.

[5] Akkawi I, Zmerly H (2018). Osteoporosis: current concepts. Joints.

[6] Woitge HW, Seibel MJ, Ziegler R (1996). Comparison of total and bone-specific alkaline phosphatase in patients with nonskeletal disorders or metabolic bone diseases. Clin Chem.

[7] Farley JR, Baylink DJ (1986). Skeletal alkaline phosphatase activity as a bone formation index in vitro. Metabolism.

[8] VanStraalen JP, Sanders E, Prummel MF, Santers GT (1991). Bone alkaline phosphatase as indicator of bone formation. Clin Chim Acta.

[9] von Elm E, Altman DG, Egger M, Pocock SJ, Gøtzsche PC, Vandenbroucke JP (2007). The Strengthening the Reporting of Observational Studies in Epidemiology (STROBE) statement: guidelines for reporting observational studies. Ann Intern Med.

[10] Laurer H, Hagenbourger O, Quast S, Herrmann W, Marzi I (2000). Sequential changes and pattern of bone-specific alkaline phosphatase after trauma. Eur J Trauma.

[11] Lowe D, Sanvictores T, John S (2022). Alkaline Phosphatase. https://www.ncbi.nlm.nih.gov/books/NBK459201/#_NBK459201_pubdet_.

[12] Hanche-Olsen TP, Alemu L, Viste A, Wisborg T, Hansen KS (2012). Trauma care in Africa: a status report from Botswana, guided by the World Health Organization's "Guidelines for Essential Trauma Care". World J Surg.

[13] Gosselin RA, Spiegel DA, Coughlin R, Zirkle LG (2009). Injuries: the neglected burden in developing countries. Bull World Health Organ.

[14] Soleymanha M, Mobayen M, Asadi K, Adeli A, Haghparast-Ghadim-Limudahi Z (2014). Survey of 2582 cases of acute orthopedic trauma. Trauma Mon.

[15] Rastogi D, Meena S, Sharma V, Singh GK (2014). Causality of injury and outcome in patients admitted in a major trauma center in North India. Int J Crit Illn Inj Sci.

[16] Chaudhary C, Singh A, Pathak R, Ahluwalia SK, Goel RK, Mithra P (2013). Predictors of seatbelt and helmet usage among victims seeking care at emergency department in a tertiary care hospital in rural Northern India. Nepal J Med Sci.

[17] Rohilla RK, Kumar S, Singh R, Devgan A, Meena HS, Arora V (2019). Demographic study of orthopedic trauma among patients attending the accident and emergency department in a tertiary care hospital. Indian J Orthop.

[18] Yessayan L, Moore C, Lu M, Yee J (2017). Bone-specific alkaline phosphatase and bone turnover in African American hemodialysis patients. Hemodial Int.

[19] Biver E, Chopin F, Coiffier G, Brentano TF, Bouvard B, Garnero P, Cortet B (2012). Bone turnover markers for osteoporotic status assessment? A systematic review of their diagnosis value at baseline in osteoporosis. Joint Bone Spine.

[20] Tariq S, Tariq S, Lone KP, Khaliq S (2019). Alkaline phosphatase is a predictor of bone mineral density in postmenopausal females. Pak J Med Sci.

[21] Garnero P, Delmas PD (1993). Assessment of the serum levels of bone alkaline phosphatase with a new immunoradiometric assay in patients with metabolic bone disease. J Clin Endocrinol Metab.

[22] Krappinger D, Kammerlander C, Hak DJ, Blauth M (2010). Low-energy osteoporotic pelvic fractures. Arch Orthop Trauma Surg.

[23] Chen H, Li J, Wang Q (2018). Associations between bone-alkaline phosphatase and bone mineral density in adults with and without diabetes. Medicine (Baltimore).

